# BAAR: A framework for blockchain-based anonymous and revocable user authentication scheme

**DOI:** 10.1371/journal.pone.0343696

**Published:** 2026-03-31

**Authors:** Muhammad Ahmed, Adnan Ahmad, Furkh Zeshan, Sheeraz Akram

**Affiliations:** 1 Computer Science Department, COMSATS University Islamabad, Lahore Campus, Lahore, Pakistan; 2 Information Systems Department, College of Computer and Information Sciences, Imam Mohammad Ibn Saud Islamic University (IMSIU), Riyadh, Saudi Arabia; University of Electronic Science and Technology of China, CHINA

## Abstract

Blockchain-based systems increasingly require authentication mechanisms that simultaneously preserve user privacy, support accountability, and enable efficient credential revocation. However, most existing anonymous authentication schemes rely on pairing-based cryptography which introduce high computational overhead and limit deploy ability on widely adopted blockchain platforms such as Ethereum. This paper presents BAAR, a Blockchain-based Anonymous and Revocable authentication framework designed entirely within the discrete logarithm setting over the secp256k1 elliptic curve. BAAR integrates Pedersen vector commitments, Schnorr-based zero-knowledge proofs, and a Merkle-tree-based dynamic accumulator to support anonymous and unlinkable authentication with selective attribute disclosure and public, auditable revocation. Authentication and proof verification are performed off-chain, while the blockchain maintains only a compact revocation state, significantly reducing on-chain computation and gas costs. A formal security analysis demonstrates unforgeability, unlinkability, attribute privacy, and revocation soundness under standard cryptographic assumptions in the random oracle model. A prototype implementation on Ethereum confirms that BAAR achieves low gas consumption, logarithmic-time revocation, and scalable performance with respect to both the number of users and attributes. These results indicate that BAAR provides a practical balance between strong privacy guarantees and deploy ability, making it suitable for real-world blockchain-based identity and access-control systems.

## 1. Introduction

User authentication is a fundamental component of modern digital security, ensuring that only authorized individuals are able to access sensitive data and digital services. Authentication factors are typically classified into three categories: knowledge-based (e.g., passwords), possession-based (e.g., tokens or smart cards), and attribute-based (e.g., biometrics such as fingerprints or facial recognition) [1,2]. While multi-factor approaches strengthen resilience against cyberattacks, large-scale data breaches — such as those affecting Facebook (2019), LinkedIn (2021), Yahoo (2014), Zoom (2020), and Baidu [3–8] — reveal that billions of user credentials and attributes remain vulnerable. Beyond credential leakage, the rise of location-based services such as Google Maps and Baidu Maps also poses risks to privacy and physical safety [9,10].

These incidents underscore the pressing need for authentication systems that offer both robust security and privacy protection. Blockchain-based systems increasingly rely on authentication mechanisms to regulate access to decentralized services and digital assets. However, existing authentication approaches face difficulty in simultaneously satisfying three competing requirements: strong user anonymity, effective credential revocation, and practical deploy ability on widely used blockchain platforms. Many privacy-preserving authentication schemes achieve anonymity through pairing-based cryptography or complex zero-knowledge constructions, which introduce substantial computational overhead and limit compatibility with standard blockchain infrastructures such as Ethereum [11,12]. As a result, these schemes are often unsuitable for real-world deployment scenarios where efficiency, scalability, and interoperability are critical.

The problem addressed in this work arises from the absence of a practical blockchain-based authentication framework that can jointly support anonymity, unlinkability, selective revocation, and compatibility with existing blockchain platforms without relying on computationally expensive cryptographic primitives [13–16]. In particular, the reliance on pairing-friendly curves and circuit-intensive proof systems in many existing designs hinders adoption on platforms that natively employ the secp256k1 elliptic curve, including Bitcoin, Ethereum, and Hyperledger Fabric [17,18]. Although recent studies have explored privacy-preserving mechanisms based on secp256k1 [19], most existing anonymous credential systems continue to depend on pairing-based constructions, limiting their scalability and deploy ability. Prior comparative analyses indicate that secp256k1, when combined with Schnorr-based protocols, offers an efficient cryptographic foundation for blockchain-oriented anonymity and authentication [20]. This study focuses on the design and evaluation of an anonymous authentication framework for blockchain-based environments. The proposed work addresses credential issuance, authentication, selective attribute disclosure, and revocation, which are central to privacy-preserving access control in decentralized systems. The framework is designed for blockchain platforms based on the secp256k1 elliptic curve, with Ethereum used as a representative deployment environment for maintaining a publicly auditable revocation state. The security analysis considers standard cryptographic assumptions relevant to anonymous authentication and credential revocation.

In relation to existing research on blockchain-based anonymous authentication, this work contributes to the literature by addressing the gap between theoretically expressive privacy-preserving schemes and frameworks that are practically deployable on widely adopted blockchain platforms. Prior studies predominantly rely on pairing-based cryptography or general-purpose zero-knowledge systems, which provide strong security guarantees but incur substantial computational and implementation complexity. In contrast, this work demonstrates how established discrete-logarithm–based primitives can be systematically combined to support anonymity, selective disclosure, and efficient public revocation within the constraints of current blockchain infrastructures. By emphasizing deploy ability alongside privacy guarantees, the proposed approach extends the applicability of anonymous authentication mechanisms to real-world blockchain systems. In response to these challenges, this paper presents the Blockchain-based Anonymous and Revocable authentication (BAAR) framework, which operates entirely in the discrete logarithm setting over the secp256k1 elliptic curve, ensuring compatibility with existing blockchain platforms [21,22]. BAAR integrates Pedersen vector commitments, Schnorr-based zero-knowledge proofs, and a Merkle-tree-based dynamic accumulator to enable anonymous and revocable authentication with selective attribute disclosure [23]. The framework achieves anonymity, unlinkability, multi-attribute credential support, and efficient public revocation while maintaining lightweight computation and practical deploy ability under standard security assumptions.

### 1.1. Contributions

The contributions of this work are summarized as follows:

1. **Practical authentication framework compatible with deployed blockchains:** This work presents a blockchain-based anonymous authentication framework that operates entirely in the discrete logarithm setting over the secp256k1 elliptic curve. The proposed design avoids pairing-based cryptography and circuit-intensive proof systems, ensuring compatibility with widely deployed blockchain platforms such as Ethereum.
2. **Anonymous and unlinkable authentication with public revocation:** The proposed framework supports anonymous and unlinkable user authentication while enabling efficient and transparent credential revocation through a Merkle-tree-based dynamic accumulator. This approach allows revoked credentials to be invalidated without revealing user identities or affecting non-revoked users.
3. **Multi-attribute credentials with selective disclosure:** The scheme supports credentials bound to multiple attributes using Pedersen vector commitments. During authentication, users can selectively disclose only the required attributes, while undisclosed attributes remain cryptographically protected.
4. **Efficient separation of off-chain verification and on-chain state management:** Schnorr-based zero-knowledge proofs and signature verification are performed off-chain, whereas the blockchain is used solely to maintain the revocation state. This separation significantly reduces on-chain computational overhead and gas consumption while preserving public auditability.
5. **Security analysis under standard cryptographic assumptions:** The security properties of the proposed framework, including unforgeability, unlinkability, attribute privacy, and revocation soundness, are analyzed under standard discrete-logarithm–based assumptions in the random oracle model.
6. **Prototype implementation and experimental evaluation:** A prototype implementation on Ethereum is developed to evaluate the practical performance of the proposed framework. Experimental results demonstrate low on-chain gas costs, logarithmic revocation overhead, and scalability with respect to the number of attributes.

The remainder of this paper is organized as follows. Section 2 reviews the related work. Section 3 introduces the necessary cryptographic preliminaries and presents the BAAR system model. Section 4 outlines the BAAR security model and formal security assumptions. Section 5 details the proposed BAAR scheme, including its design and operational phases. Section 6 presents the model implementation and evaluates the performance of the scheme in terms of computational overhead, gas consumption, revocation efficiency, and scalability. Section 7 concludes the paper.

## 2. Literature review

This section discussed the anonymous authentication schemes with an emphasis on anonymity, revocation, efficiency, and suitability for blockchain applications. While many solutions have been proposed which most suffer from high computational cost, weak revocation, limited scalability, and motivating the need for more practical designs.

Early research work as reported in literature [24–27,28,29] explored the application of randomization methods and identity based cryptographic primitives as a means of achieving anonymity. However, they faced significant challenges in scalability, attribute privacy, and revocation, limiting their applicability in decentralized environments. Wang et al. [30] extended the identity mixer framework [31] with zero-knowledge proofs to allow selective disclosure of attributes. It improved privacy but it did not offer full anonymity with efficient revocation mechanisms. The authors [32] suggested a computationally efficient and pairing -free aggregate signature scheme, which is especially applicable for blockchain. But it suffered of high computational costs and reliance on third-party. Khalid et al. [33] proposed an authentication system based on blockchain. The articulated technique resolved the problems involved with existing remote authentication techniques. The suggested model did not help to alleviate scalability problems. Bisht et al. [34] proposed a revocation system of anonymous credentials using a threshold on blockchain as a means of reducing the revocation inefficiency of earlier systems. Sonnino et al. [35] presented a selective disclosure credential scheme including blockchain technology to maintain secret and authenticity, but this scheme lacks the revocation mechanism, which is considered a critical aspect to implement in the real world environment. Yang et al. [13] presented an aggregation signcryption scheme without certifies and anonymity which is proposed to be used in Vehicular Ad Hoc Network (VANET) safety warning systems. Although the scheme has the benefit of preserving privacy, it requires the assistance of a trusted authority and expensive bilinear pairings. Therefore, it has limiting scalability in large, real-time settings. Wang et al. [14] proposed a 6G revocation system that uses the RSA multi-accumulators and blockchain technology to optimize the latency and load balancing. The main disadvantage of this method is that it has a high computational cost, thus it is not applicable in lightweight blockchain systems. Wang and Zhang [15] proposed a selective disclosure model, which uses Merkle-based data structure and zero-knowledge Succinct Non-Interactive ARguments of Knowledge (zkSNARKs) to safeguard identity data. The scheme was later generalized to multi-attribute credentials but the cost of generating zk-SNARKs, combined with the cost scaling with the number of attributes of maintaining the Merkle paths which leading to higher validation latency. Yu et al. [16] proposed a system that combines dynamic accumulators, digital signatures, and zero-knowledge proofs with the help of a blockchain ledger. Although this technique supports selective revocation and multi-attribute privacy, the multi-round nature of the protocol provisional cost is significant. Yang et al. [36] introduced Zero-Cerd which was a self-blindable anonymous authentication system that uses dynamic accumulators. Even though there is a reduction in privacy and linkability in the scheme, it has a high overhead in terms of communication and computation. Shi et al. [37] introduced the concept of selective disclosure that replaces zero-knowledge proofs with unlinkable redactable signatures based on polynomials constructions, which lowers the cost of disclosure computation. However, this strategy is not concerned with the effectiveness of operations in the underlying chain. Ahmed et al. [38] proposed a shard-chain blockchain system that is aimed at increasing scalability and efficiency, with the distribution of transactions and smart contracts between parallel chains. The strategy aims at reducing the transaction latency and storage overhead and at the same time enhancing the overall system performance in Ethereum-based environments. Khan et al. [39] suggested a lightweight and scale able hybrid authentication infrastructure of the Internet of Medical Things (IoMT) settings, combining the Hyperledger Indy consortium blockchain with edge computing. The protocol alleviates the efficiency of authentication and minimizes the latency by implementing permission blockchain infrastructures and proxy-based cryptographic schemes. Xiong et al. [40] proposed an attribute-based encryption scheme to enable fine-grained access-control for digital twins with revocable feature. The scheme concentrates on the data confidentiality and it is based on the pairing cryptography. Wang et al. [41] introduced the proxy re-encryption scheme of the safe cross-system information sharing in clouds. The design team is geared towards the encrypted data management where the structures are built using pairing-based technique. Existing blockchain-based anonymous authentication systems present a wide range of cryptographic features in order to support privacy, revocation and accountability. Even though the given methodologies offer strong security guarantees, many of them focus on generality or expressive proof systems, which makes them have more severe computational overheads or less compatibility with prevalent blockchain infrastructures. In this context, BAAR framework is placed as a viable option that highlights deploy ability under the limitation of modern blockchain platforms. The BAAR system, using only discrete-logarithm based primitives, with a Merkle tree-based revocation scheme, is an example of how well-known cryptographic mechanisms can be designed to provide such properties as anonymity, selective disclosure, and revocation on a large scale. The role of BAAR towards this positioning is that it is a consistent framework on which viable deployment parameters can be based in tandem with security guarantees.

As Table 1 indicates, no prior work simultaneously provides (i) pairing-free operation on secp256k1, (ii) multi-attribute selective disclosure with lightweight zero knowledge, and (iii) public, logarithmic-time revocation suitable for on-chain publication. BAAR fills this gap by combining Pedersen vector commitments, Schnorr signature, and a Merkle-based dynamic accumulator, yielding an efficient and deployable construction without pairings.

**Table 1:** Comparison of Literature Review with BAAR.

Scheme	Deactivation	Identity Privacy	Distributed Ledgers	Revocation	Unlinkability	Running Time
[24]	✗	✓	✗	✗	✓	O(n)
[25]	✗	✓	✗	✓	✗	O(1)
[26]	✗	✓	✗	✓	✗	O(n)
[27]	✗	✓	✗	✗	✗	O(1)
[30]	✗	✓	✗	✗	✗	O(1)
[31]	✗	✓	✓	✗	✓	O(n)
[32]	✗	✗	✓	✗	✗	O(n)
[33]	✗	✗	✓	✗	✗	O(n)
[34]	✓	✓	✓	✓	✓	O(n2)
[35]	✗	✓	✗	✓	✗	O(n)
[13]	✓	✓	✗	✓	✗	O(n)
[14]	✗	✓	✓	✓	✓	O(n)
[15]	✗	✓	✓	✗	✓	O(n log n)
[16]	✗	✓	✓	✓	✓	O(n)
[36]	✗	✓	✓	✓	✓	O(n)
[37]	✗	✓	✓	✓	✓	O(n log n)
[38]	✗	✗	✗	✗	✗	O(log n)
[39]	✗	✗	✗	✗	✗	O(log n)
[40]	✗	✓	✗	✓	✗	N/A
[41]	✗	✓	✗	✗	✗	N/A
BAAR	✓	✓	✓	✓	✓	O(logn)

✓: represent the Yes. ✕: represent the No.

## 3. Preliminaries and BAAR system

This section introduces the cryptographic preliminaries and system entities used in BAAR. The system first defines mathematical assumptions and then presents the overall proposed system model Table 2.

**Table 2:** Notations used in BAAR.

Symbol	Description
q	Large prime order of the elliptic-curve group.
𝔾	Elliptic-curve group of order q (e.g., secp256k1).
B	Base point (generator) of 𝔾.
d	User’s private key, sampled uniformly from $Z_{q}$
p	Prime modulus of secp256k1 finite field
$F_{p}$	Elliptic curve over field $F_{p}$
n	Order of the subgroup generated by G
h	Cofactor of the elliptic curve group
d	User’s private key (scalar in $Z_{q}$)
e=d·B	User’s public key (point on the curve)
U,V	Parties (User A, Verifier B)
m	Message to be signed/verified
σ	Digital signature
$\left\{m_{i}\right\}$	Set of messages in an aggregate signature
$a=a_{\text{1}},\ldots ,a_{m}$	User’s attribute vector with m attributes.
τ	Token/transaction identifier
$x_{A},x_{B}$	Secret exponents of User A and Verifier B
$y_{A},y_{B}$	Public values derived from secret exponents
r	Random nonce used in commitments
c	Challenge in Schnorr ZKPoK
s	Response in Schnorr ZKPoK
π	Zero-knowledge proof transcript
H(·)	Collision-resistant hash function
$w_{i}$	Membership witness for credential i
O	Random oracle
κ	Security parameter/challenge string
*Tᵢ*	Independent generator for the i-th attribute commitment.
*r*	Random blinding factor in ℤ_q.
C	Pedersen commitment to attribute(s)
*C = r·B + Σ aᵢ·Tᵢ*	Pedersen vector commitment to attribute vector a.
$k_{r}$, $k_{i}$	Random values used when constructing ZK proofs.
*R*	Commitment in Schnorr/ZK protocol (e.g., R = k_r·B + Σ kᵢ·Tᵢ).
$z_{r}$, $z_{i}$	ZK response values for blinding and attribute components.
*ZKPoK*	Zero-Knowledge Proof of Knowledge (Σ‑protocol proving the opening of C).
*σ = (R, s)*	Schnorr signature on message m.
s=k+c.d	Schnorr signature response component.
H(.)	Collision-resistant hash function (e.g., SHA‑256).
*x = H(“tag”* ∥ *C)*	Identifier derived from C for use as a Merkle-accumulator leaf.
Acc	Current Merkle accumulator root representing valid credentials.
Acc′	Updated accumulator root after revocation
*MerkleVerify(Acc, x, π)*	Verification function that checks Merkle membership.
$d_{u}$, $e_{u}$	Temporary user key pair used during credential request.
*π′*	Selective disclosure ZK proof used during presentation.
S ⊆{1,….,m}	Index set of attributes to be disclosed.
*S*	Complement of S — the set of hidden attributes.
*λ*	Security parameter.
A	Adversary

### 3.1. Preliminaries

All cryptographic primitives in BAAR are instantiated over the secp256k1 elliptic-curve group G=⟨B⟩ of prime order q. This ensures efficiency and compatibility with widely deployed blockchain ecosystems such as Ethereum. In BAAR, the security of all core mechanisms is grounded in the hardness of the elliptic-curve discrete logarithm problem (ECDLP) on secp256k1 [42]. Specifically, Schnorr signatures for authentication, Pedersen commitments for attribute binding, and the associated ZKPoK are all constructed within this discrete-logarithm setting.

A. **System Parameters**

BAAR is instantiated over the secp256k1 elliptic curve defined by parameters (p,a,b,h,q,B), where p is the prime modulus and (a,b) define the curve equation $y^{\text{2}}=x^{\text{3}}+ax+b\;\;mod\;p$. The base point $B=(x_{B},y_{B})$ generates a subgroup of prime order q with cofactor h. All cryptographic operations are carried out in this subgroup, and the security of the scheme relies on the hardness of the ECDLP. To support multi-attribute credentials, the system additionally selects a set of independent generators $\left\{T_{\text{1}},T_{\text{2}},\ldots ,T_{m}\right\}$, where m denotes the maximum number of attributes per credential. Together with the blinding generator B, these constitute the public parameters for Pedersen vector commitments [43]. For an attribute vector $𝐚=(a_{\text{1}},\ldots ,a_{m})$, the credential commitment is computed as


$$C=rB+\sum_{i=\text{1}}^{m}a_{i}T_{i}$$ (1)

where $r\in Z_{q}$ is a random blinding factor. This binding forms the cryptographic basis for selective disclosure and zero-knowledge proofs in BAAR.

B. **Assumption**

Let p be a prime modulus, and let the elliptic curve over $F_{p}$ be defined by the equation $y^{\text{2}}=x^{\text{3}}+ax+b(\text{mod}p)$. Let G=⟨B⟩ be the subgroup generated by the base point B, with prime order q and cofactor h. We assume the hardness of the ECDLP: given X=xB for uniformly random $x\in Z_{q}$, no probabilistic polynomial-time adversary can recover x. All hash functions $H:\{\text{0},\text{1}{\}}^{*}\to Z_{q}$ are modelled as random oracles, i.e., idealized functions that output uniformly random values in $Z_{q}$.

C. **Pedersen Vector Commitments and Zero-Knowledge Proofs**

BAAR employs Pedersen vector commitments to protect user attributes. For an attribute vector $𝐚=(a_{\text{1}},a_{\text{2}},\ldots ,a_{m})\in \overset{m}{\underset{q}{Z}}$ and a random blinding factor $r\in Z_{q}$, the commitment is defined as equation 1.


$$C=rB+\sum_{i=\text{1}}^{m}a_{i}T_{i}$$

where B is the blinding generator and $T_{\text{1}},\ldots ,T_{m}$ are independent generators for each attribute. These commitments are perfectly hiding, as they reveal nothing about the attribute values and binding, since opening to different values would require solving the ECDLP on secp256k1. To prove the correctness of a commitment without revealing the attributes, BAAR employs ZKPoK of the form.


$$\text{ZKPoK}\{(r,a_{\text{1}},\ldots ,a_{m}):C=rB+\sum_{i=\text{1}}^{m}a_{i}T_{i}\}$$ (2)

This proof follows a standard Σ-protocol made non-interactive using the Fiat–Shamir heuristic [44], resulting in short transcripts that are efficient to verify. Crucially, the protocol supports selective disclosure: the prover may reveal a chosen subset of attributes while proving knowledge of the remaining hidden ones. This enables fine-grained privacy and unlinkability while maintaining lightweight verification on the secp256k1 curve.

D. **Schnorr Signatures**

BAAR utilizes Schnorr signatures over the secp256k1 curve for user authentication. A user selects a secret key $x\in Z_{q}$ with the corresponding public key Y=xB. To sign a message m, the signer chooses a random nonce $k\in Z_{q}$, computes the commitment R=kB, derives the challenge c=H(R∥m), and outputs the response s=k+cx(modq), forming the signature σ=(R,s). Verification consists of recomputing c=H(R∥m) and checking sB\stackrel?=R+cY. Security follows from the hardness of the ECDLP in the random oracle model.

E. **Dynamic Accumulator**

For credential revocation, BAAR employs a hash-based dynamic accumulator implemented as a Merkle tree, instantiated with a collision-resistant hash function $H:\{\text{0},\text{1}{\}}^{*}\to \{\text{0},\text{1}{\}}^{\lambda }$. Each credential identifier $x_{i}$ is mapped to a leaf $L_{i}=H(\text{tag}∥x_{i})$, and the accumulator value is defined as the Merkle root $\text{Acc}=\text{MerkleRoot}(L_{\text{1}},\ldots ,L_{n})$. A membership witness for credential $x_{j}$ is the authentication path $\pi_{j}\;$consisting of sibling hashes up to the root, and verification checks whether the recomputed root satisfies $\text{MerkleVerify}(x_{j},\pi_{j})\mathrm{\backslash stackrel}?=\text{Acc}$. Dynamic updates, such as adding or revoking a credential, require modifying only O(logn) nodes along the affected path. At the same time, all other witnesses remain valid except for siblings on that path. Security relies on the collision resistance of H: valid members can always reconstruct the root, whereas non-members cannot forge valid paths without breaking the hash assumption. This provides efficient selective revocation and integrates naturally with BAAR’s ZKPoK over the secp256k1 curve. Prior works [45–47] confirm the efficiency of Merkle trees as accumulators, demonstrating their suitability in discrete logarithm-based environments.

### 3.2. BAAR system

**Fig 1:**
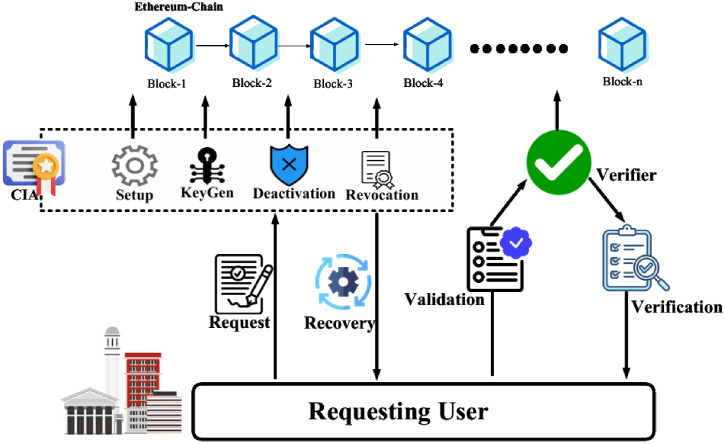
BAAR Scheme with Entities.

The BAAR model comprises four entities: the blockchain, the Credential Issuing Authority (CIA), verifiers, and users, as shown in Fig 1.

1. Blockchain: A public ledger that publishes system parameters, public keys, and the current Merkle accumulator root for credential validity.
2. CIA: Initializes the system by generating keys and parameters, issues Pedersen-based credential commitments to users (supporting both single and multi-attribute credentials), and updates the accumulator root to reflect revocation.
3. Verifiers: Retrieve the latest accumulator root from the blockchain and validate user credentials using Schnorr signatures and ZKPoK proofs.
4. Users: Obtain system parameters, request credentials from the CIA, and later prove membership and attributes to verifiers. In the multi-attribute setting, users may selectively disclose only the required subset of attributes while keeping the rest hidden. Upon revocation, users coordinate with the CIA to refresh their Merkle witnesses.

### 3.3. BAAR syntax for anonymous authentication

An anonymous authentication scheme with selective revocation consists of the following steps:

- **Setup** $({\text{1}}^{\lambda })\to par$: Takes the security parameter λ and outputs the public system parameters par.
- **KeyGen** (par)→(d,e): Each user samples a private key $d\in Z_{q}$ and computes the public key e=d·B. The CIA initializes the on-chain Revoked Credential List RCL=∅.
- **Issue** (par,a,e)→σ: An interactive protocol between a user and the CIA. The user commits to a vector of attributes $\text{a}=(a_{\text{1}},a_{\text{2}},\ldots ,a_{m})$ and proves possession of d. The CIA issues a credential σ without learning the values of a.
- **Request** (par,σ)→Θ: The user generates a non-interactive ZKPoK Θ of credential possession, together with a Merkle membership witness. In the multi-attribute setting, the proof supports the selective disclosure approach.
- **Verify** (par,e,Θ)→b: The verifier checks proof Θ under public key e; outputs b=1 if valid and otherwise b=0.
- **Deactivate** $(par,RCL)\to RCL^{\prime }$: The CIA deactivates a credential by adding its identifier to the revocation list and publishing the updated root $RCL^{\prime }$on-chain.
- **Recover** $(par,e)\to \sigma_{\text{new}}$:A user can obtain a fresh credential through re-issuance if the old one is lost or expired.
- **Revoke** $(par,e,\Theta ,RCL)\to RCL^{\prime }$: The CIA revokes a credential by updating the revocation list and publishing the new root $RCL^{\prime }$on-chain.

### 3.4. BAAR security model

#### 3.4.1. Security assumptions.

BAAR is analyzed in the random oracle model (ROM) over the secp256k1 elliptic-curve group G=⟨B⟩ of prime order q.

- ***A1 – Discrete Logarithm Hardness:*** Given a generator B∈G and a public key e=dB for some secret $d\in Z_{q}$, no probabilistic polynomial-time adversary can recover d with non-negligible probability.
- ***A2 – Schnorr Signature Security:*** Schnorr signatures are existentially unforgeable under chosen-message attacks (EUF-CMA) in the ROM, assuming the hardness of ECDLP.
- **A3 – ZKPoK Security:** ZKPoK for Pedersen commitments (including vector commitments over multiple attributes), when made non-interactive via the Fiat–Shamir heuristic, are sound and zero-knowledge in the ROM.
- ***A4 – Merkle Accumulator Soundness:*** No adversary can produce a valid membership witness (x,π) for an element x∉X without breaking the collision resistance of the hash function H.
- ***A5 – Hash Function Security:*** All hash functions used in Schnorr signatures, Pedersen commitments, and Merkle accumulators are modeled as random oracles and assumed to be collision-resistant.

#### 3.4.2. Security games.

BAAR defines its security through the following standard indistinguishability games between a challenger C and an adversary A.

1- ***Unforgeability:*** The adversary A interacts with an issuing oracle to obtain credentials of its choice. Finally, it outputs a transcript τ. The game outputs 1 if verify(τ)=1 for a credential not issued to A. Formally,

$$\overset{UF}{\underset{A}{\text{Adv}}}(\lambda )=\text{Pr}[\text{Forge}(A)=\text{1}]\leq \text{negl}(\lambda ).$$

Security reduces to the unforgeability of Schnorr signatures under ECDLP and the soundness of Pedersen-based ZKPoK. In the multi-attribute setting, unforgeability additionally ensures that an adversary cannot create a valid credential for an attribute vector "a" it was never issued.

2- ***Credential Soundness:*** Let Acc denote the Merkle root of the set of valid credentials X. The adversary wins if it produces a witness (x,π) such that;

MerkleVerify(Acc,x,π)=1for x∉X.

The advantage of A is negligible under the collision resistance of the hash function H.

3- ***Unlinkability:*** The challenger generates credentials for two users $U_{\text{0}}$ and $U_{\text{1}}$. It chooses a hidden bit b∈{0,1} and outputs an authentication transcript for $U_{b}$. The adversary outputs a guess $b^{\prime }$. The unlinkability advantage is defined as

$$\begin{array}{c} \overset{UL}{\underset{A}{\text{Adv}}}(\lambda )=|\text{Pr}[b=b^{\prime }]-\frac{\text{1}}{\text{2}}|\leq \text{negl}(\lambda ). \end{array}$$

This property follows from the hiding of Pedersen commitments and the ZKPoK property of the proofs, which ensure that transcripts cannot be linked across sessions. In the multi-attribute case, unlinkability further guarantees that revealing one attribute does not compromise the privacy of undisclosed attributes.

4- ***Revocation Soundness:*** After a credential is revoked and the Merkle accumulator root updated, the adversary must not be able to produce a valid transcript τ corresponding to the revoked credential. Formally,

$$\begin{array}{c} \text{Pr}[\text{Verify}(\tau )=\text{1}|\tau \text{ corresponds to a revoked credential}]\leq \text{negl}(\lambda ). \end{array}$$

#### 3.4.3. Security argument.

BAAR achieves its security objectives under standard cryptographic assumptions, including unforgeability, credential integrity, attribute privacy, credential deactivation, selective revocation, and unlinkability.

1- ***Unforgeability:*** Only legitimate users holding valid credentials can generate authentication transcripts. Forging a transcript without a valid credential contradicts the existential unforgeability of Schnorr signatures$\overset{UF}{\underset{A}{\text{Adv}}}(\lambda )\leq \text{negl}(\lambda ).$
2- ***Credential Integrity:*** A verifier cannot be convinced of a credential that does not exist. This follows from Merkle accumulator soundness under the collision resistance of H.
3- **Attribute Privacy.** The CIA and verifiers learn nothing beyond explicitly disclosed attributes. In the multi-attribute setting, hidden attributes remain protected by Pedersen commitments and ZKPoK.
4- **Credential Deactivation.** Once revoked, a credential cannot be reused for authentication since verification will fail against the updated accumulator root.
5- **Selective Revocation.** The CIA can revoke credentials by updating the Merkle root without attribute disclosure. Any attempt to use a revoked credential succeeds only with negligible probability.
6- **Unlinkability.** Multiple presentations of the same credential cannot be correlated. Even in the multi-attribute setting, transcripts remain unlinkable even if some attributes are selectively disclosed $\overset{UL}{\underset{A}{\text{Adv}}}(\lambda )\leq \text{negl}(\lambda ).$

### 3.5. Multi-attribute security

The same security arguments apply to the multi-attribute setting, as the scheme was designed to support multiple attributes from the outset. Pedersen vector commitments preserve hiding and binding regardless of the number of attributes, ensuring that undisclosed attributes remain private while preventing forgery. The Schnorr-based ZKPoK is likewise extensible: users can jointly prove knowledge of all committed attributes or selectively disclose only a subset, without compromising soundness. Since each multi-attribute credential is a single entry in the Merkle accumulator, revocation remains unaffected and integrity is preserved.

Formally, let $\text{a}=(a_{\text{1}},\ldots ,a_{m})$ be a vector of attributes. The Pedersen commitment is:


$$C=\overset{a_{\text{1}}}{\underset{\text{1}}{g}}\overset{a_{\text{2}}}{\underset{\text{2}}{g}}\cdots \overset{a_{m}}{\underset{m}{g}}h^{r}$$

To prove knowledge, the user samples $\left(w_{\text{1}},\ldots ,w_{m},w_{r}\right)$ and computes:


$$T=\overset{w_{\text{1}}}{\underset{\text{1}}{g}}\overset{w_{\text{2}}}{\underset{\text{2}}{g}}\cdots \overset{w_{m}}{\underset{m}{g}}h^{w_{r}}$$

Given a challenge $c\in Z_{q}$, responses are:


$$s_{i}=w_{i}+ca_{i}(i=\text{1},\ldots ,m),\;s_{r}=w_{r}+cr$$

Verification succeeds if:


$$\overset{s_{\text{1}}}{\underset{\text{1}}{g}}\overset{s_{\text{2}}}{\underset{\text{2}}{g}}\cdots \overset{s_{m}}{\underset{m}{g}}h^{s_{r}}\;\;\mathrm{\backslash stackrel}?=\;\;T\cdot C^{c}$$ (3)

The equation (3) satisfies completeness and soundness: honest users pass, adversaries cannot forge consistent responses, and simulated transcripts leak nothing. Thus, BAAR supports multi-attribute credentials by design, without requiring additional cryptographic assumptions or complexity.

**Details of BAAR Scheme:**

The operation of BAAR is divided into four main phases—Request, Generation, Validation, and Verification—which collectively define the end-to-end lifecycle of anonymous and revocable user authentication, as illustrated in Fig 1.

1. ***Request Phase:*** In the request phase, a user initializes the process by sampling a key pair (d,e=d·G) and preparing an attribute vector $m=(m_{\text{1}},\ldots ,m_{l})$. The user then generates a temporary key pair $\left(d_{u},e_{u}\right)$ and computes a Pedersen vector commitment to the attribute vector,

$$\begin{array}{c} C=r\cdot H+\sum_{i=\text{1}}^{\text{𝓁}}m_{i}\cdot G_{i},r\leftarrow Z_{q} \end{array}$$ (4)

Using a Schnorr-based Σ-protocol (Fiat–Shamir transformed), the user produces a ZKPoK π demonstrating knowledge of the committed attributes and the blinding factor without revealing them. Finally, the user sends the credential issuance request to the CIA.


$$\text{Request}=\left(e_{u},C,\pi ,{\text{Sign}}_{d_{u}}(C)\right)$$

2. ***Generation Phase:*** Upon receiving the request, the CIA verifies both the ZKPoK π and the signature under $e_{u}$. If valid, the CIA inserts the commitment C into the Merkle accumulator, updating the root as

$$\overset{\prime }{\underset{\text{root}}{\text{Acc}}}=\text{Merkle}.\text{Insert}(C,{\text{Acc}}_{\text{root}})$$

The updated accumulator root is published on-chain to ensure global verifiability, and the corresponding Merkle membership witness w is returned to the user. At this point, the credential is officially issued and registered.

3. ***Validation Phase:*** In the validation phase, the user prepares for authentication. A subset of attributes S⊆{1,…,𝓁} is selected for disclosure, while the remaining attributes stay hidden. The user constructs a selective-disclosure ZKPoK $\pi^{\prime }$ attesting to the committed but undisclosed attributes, and signs the proof and Merkle witness with their temporary private key, producing

$$\sigma =\text{Schnorr}.\text{Sign}(d_{u},\pi^{\prime }∥w)$$

The user then sends $\left(C,e_{u},m_{S},\pi^{\prime },w,\sigma \right)$ to the verifier.

4. ***Verification Phase:*** The verifier performs three checks to validate the authentication:
  1. **Signature verification:** ${\text{Verify}}_{\text{Schnorr}}(e_{u},\sigma )=\text{1}$.
  2. **ZKPoK verification:** $\text{Verify}(\pi^{\prime },m_{S},C)=\text{1}$.
  3. **Accumulator membership verification:** $\text{Merkle}.\text{Verify}(w,C,{\text{Acc}}_{\text{root}})=\text{1}$.

If all checks succeed, the verifier accepts the authentication. This phase ensures that only valid, non-revoked credentials are accepted, while preserving user anonymity and selective disclosure.

5. ***Revocation (for completeness):*** On revocation of

$$C:\overset{\prime \prime }{\underset{\text{root}}{\text{Acc}}}=\text{Merkle}.\text{Remove}(C,\;{\text{Acc}}_{\text{root}}),$$

publish ${\text{Acc}}_{\text{root}}$ on-chain. Any future proof using C fails. Merkle.Verify(·)=1 against the updated $\overset{\prime \prime }{\underset{\text{root}}{\text{Acc}}}\;$.

#### 3.5.1. Protocol correctness.

**Protocol 1: ZKPoK for Pedersen Vector Commitments (Credential Issuance):** A user proves knowledge of committed attributes without revealing them. Let the attribute vector be $a=(a_{\text{1}},\ldots ,a_{m})$ and the commitment


$$C=rB+\sum_{i=\text{1}}^{m}a_{i}T_{i}.$$

The user chooses kr,k1,…,km←RZq and computes


$$R=k_{r}B+\sum_{i=\text{1}}^{m}k_{i}T_{i},$$

sending R to the CIA. The CIA chooses a random challenge c←RZq. The user responds with $z_{r}=k_{r}+cr\;mod\;q,z_{i}=k_{i}+ca_{i}\;mod\;q(i=\text{1},\ldots ,m).$ Verification succeeds if


$$z_{r}B+\sum_{i=\text{1}}^{m}z_{i}T_{i}\mathrm{\backslash stackrel}?=R+cC$$ (5)

Equation (5) for honest users, this holds by construction.

**Protocol 2: Schnorr Signature-Based Authentication:** For authentication, BAAR uses Schnorr signatures over secp256k1. A user with a secret d and public e=dB signs message m:

- Pick k←RZq, compute R=kB
- Compute c=H(R∥m)
- Output s=k+cdmodq, signature σ=(R,s).

Verification recomputes c and checks: sB\stackrel?=R+ce. Equality holds for honest signers, ensuring correctness.

**Protocol 3: Revocation via Merkle Accumulator:** Each credential commitment C is mapped to a leaf identifier; x=H(``tag''∥C). Leaves form a Merkle tree with a root Acc, representing all valid credentials. To revoke, the CIA removes x and publishes the new root ${\text{Acc}}^{\prime }=\text{MerkleRoot}(X\setminus \{x\}).$ During authentication, the user must present a Merkle witness π verifying


$$\text{MerkleVerify}\left({\text{Acc}}^{\prime },x,\pi \right)\mathrm{\backslash stackrel}?=\text{1}$$ (6)

**Fig 2:**
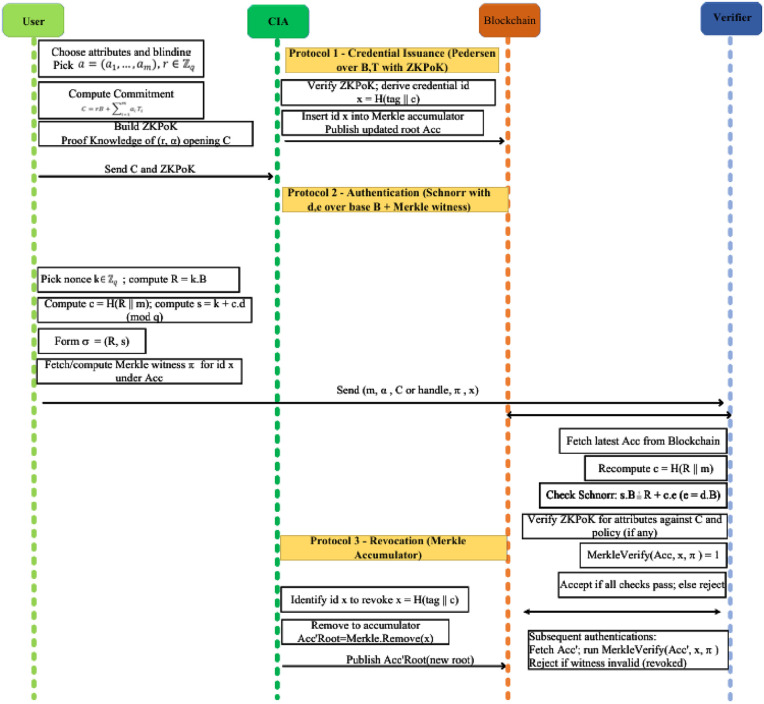
End-to-end sequence of the BAAR protocols for anonymous and revocable user authentication.

If revoked as shown in equation (6), no valid π exists. Forging one would require breaking hash collision resistance, ensuring transparent and public revocation. The overall communication flow of the protocol 1–3 as shown in Fig 2.

### 3.6. Security analysis

The BAAR scheme achieves the core security goals of unforgeability, credential soundness, attribute privacy, anonymity, and selective revocation. These properties are grounded in the hardness of the ECDLP, the unforgeability of Schnorr signatures [22], and the collision-resistance of the underlying hash functions used in Pedersen commitments and the Merkle accumulator [23]. In particular, no probabilistic polynomial-time (PPT) adversary can produce a valid authentication transcript without possessing a credential issued by the authority, thereby ensuring the system’s unforgeability.

***Lemma 1 (Unforgeability). No PPT adversary can produce a valid authentication transcript without possessing a credential issued by the authority.***

***Proof:*** Suppose an adversary A can output a valid transcript without a credential. Then, in particular, it must forge a Schnorr signature under some public key e=dB without knowing the secret d. In the Schnorr scheme, the signer selects $k\in Z_{q}$, computes R=kB, derives c=H(m∥R), and outputs s=k+cd(modq). Verification succeeds if


sB\stackrel?=R+ce.

If A produces two valid transcripts (R,c,s) and $\left(R,c^{\prime },s^{\prime }\right)$ for the same R but distinct challenges $c\neq c^{\prime }$, then the secret key can be extracted as

$d=\frac{s-s^{\prime }}{c-c^{\prime }}(\text{mod}q)$, Breaking the elliptic-curve discrete logarithm assumption. Since ECDLP is hard on secp256k1, the probability of A succeeding is negligible. Therefore, Schnorr signatures remain existentially unforgeable under chosen-message attacks (EUF-CMA) in the random oracle model, and BAAR inherits this unforgeability.

***Lemma 2 (Credential Soundness). No PPT adversary can authenticate with a credential that was never issued.***

***Proof:*** Let Acc be the Merkle root of the set of issued credentials $X=\{x_{\text{1}},\ldots ,x_{n}\}$. A valid membership proof consists of a pair (x,π) such that


MerkleVerify(Acc,x,π)\stackrel?=1

If x∉X but π is accepted, then the adversary has constructed a Merkle path that maps a non-member element x to the published root Acc. This requires producing two distinct input pairs $\left(m_{\text{1}},m_{\text{2}}\right)$ and $\left(\overset{\prime }{\underset{\text{1}}{m}},\overset{\prime }{\underset{\text{2}}{m}}\right)$ with


$$H\left(m_{\text{1}}∥m_{\text{2}}\right)=H\left(\overset{\prime }{\underset{\text{1}}{m}}∥\overset{\prime }{\underset{\text{2}}{m}}\right),$$

which is a collision in the hash function H. Since H is assumed to be collision-resistant; the probability that a PPT adversary succeeds is negligible. Therefore, no adversary can authenticate with an unissued credential.

***Lemma 3 (Attribute Privacy). BAAR preserves attribute privacy under the hiding property of Pedersen vector commitments and the zero-knowledge property of their Proofs:*** BAAR preserves attribute privacy under the hiding property of Pedersen vector commitments and the ZKPoK property of their proofs.

*Proof.* A credential is committed as


$$C=rB+\sum_{i=\text{1}}^{m}a_{i}T_{i},$$

where r is random. Pedersen vector commitments are perfectly hiding, so C reveals nothing about the attributes $\left(a_{\text{1}},\ldots ,a_{m}\right)$. To prove correctness, the user provides


$$\text{ZKPoK}\{(r,a_{\text{1}},\ldots ,a_{m}):C=rB+\sum_{i=\text{1}}^{m}a_{i}T_{i}\},$$

which can be simulated without knowledge of the attributes. Hence, transcripts leak no information beyond explicitly disclosed attributes, and hidden attributes remain private.

***Lemma 4 (Anonymity). BAAR ensures that an adversary cannot link two valid authentication transcripts to the same user, even with multi-attribute credentials.***

***Proof:*** Each authentication uses a Schnorr signature σ=(R,s) with fresh randomness $k\in Z_{q}$, where R=kB, c=H(R∥m), and s=k+cdmodq. Since R is uniformly distributed in G and independent of the secret key d, transcripts (R,c,s) are indistinguishable across sessions. Attribute commitments


$$C=rB+\sum_{i=\text{1}}^{m}a_{i}T_{i}$$

Also employ fresh randomness r, and their ZKPoK can be simulated without the attributes. Selective disclosure reveals only chosen attributes while preserving the privacy of hidden ones. Thus, the joint transcript of Schnorr signatures, ZK proofs, and Merkle membership witnesses reveals nothing that allows linking across sessions. Formally, for any adversary A:


$$\begin{array}{c} {\text{Adv}}^{UL}(A)=|\text{Pr}[b^{\prime }=b]-\frac{\text{1}}{\text{2}}|\leq \text{negl}(\lambda ). \end{array}$$

Hence, BAAR achieves unlinkability.

***Lemma 5 (Revocation Soundness). No PPT adversary can authenticate using a revoked credential.***

***Proof:*** Let Acc be the current Merkle root of all valid credentials and let C be a credential commitment that has been revoked. The CIA updates the accumulator root to ${\text{Acc}}^{\prime }$ after removing C. For authentication, a user must provide a membership witness π such that


$$\text{MerkleVerify}\left({\text{Acc}}^{\prime },C,\pi \right)\;\;\mathrm{\backslash stackrel}?=\;\;\text{1}$$

If C is revoked, no valid witness π exists. To succeed, an adversary must forge π, which requires producing a hash collision in the Merkle tree construction. Since the hash function H is assumed to be collision-resistant, the probability of success is negligible. Thus, any attempt to authenticate with a revoked credential fails with overwhelming probability.

***Lemma 6 (Authority-Forgery Resistance). A malicious authority cannot undetectably forge credentials or manipulate accumulator parameters.***

***Proof:*** Consider two attack types:

i. ***Credential forgery:*** To authenticate as a user, the authority would need a valid transcript including a Schnorr signature under the user’s public key e=dB. Producing such a signature without d contradicts Lemma 1 (Unforgeability) (EUF-CMA of Schnorr under ECDLP). Hence, impersonation is infeasible except with negligible probability.

ii. ***Accumulator manipulation:*** Let Acc be the published Merkle root for the set $X=\{x_{\text{1}},\ldots ,x_{n}\}$. If the authority publishes a tampered root ${\text{Acc}}^{\prime }\neq \text{Acc}$ to make an unissued/revoked element x∉X verify, it must provide π such that

$$\text{MerkleVerify}({\text{Acc}}^{\prime },x,\pi )\;\;\mathrm{\backslash stackrel}?=\;\;\text{1}.$$

This yields a Merkle path inconsistent with the original leaves, implying a hash collision in the tree. Since H is collision-resistant, the success probability is negligible. Moreover, because Acc is publicly posted, any divergence ${\text{Acc}}^{\prime }\neq \text{Acc}$ is detectable: honest users’ existing witnesses fail against ${\text{Acc}}^{\prime }$, exposing the manipulation. Therefore, the authority cannot forge user credentials or alter the accumulator without detection, except with negligible probability

***Lemma 7 (Selective-Disclosure Soundness & Privacy). Given a credential commitment***


$$C=rB+\sum_{i=\text{1}}^{m}a_{i}T_{i},$$

***for any disclosed index set*** S⊆{1,…,m} ***and hidden set*** $\bar{S}$***, no PPT adversary can (i) produce values*** $\{{\tilde{a}}_{i}{\}}_{i\in S}$ ***and a proof that verifies against*** C ***unless*** ${\tilde{a}}_{i}=a_{i}$ ***for all*** i∈S***, nor (ii) learn any information about*** $\{a_{i}{\}}_{i\in \bar{S}}$ ***beyond what is computationally implied by the disclosure.***

***Proof (sketch):***For (i) (soundness), the verifier checks a ZKPoK of knowledge of $\left(r,\{a_{i}{\}}_{i\in \bar{S}}\right)$ with $C^{\prime }=C-\sum_{i\in S}a_{i}T_{i}=rB+\sum_{i\in \bar{S}}a_{i}T_{i}$. By Σ-protocol soundness, any accepting proof implies knowledge of witnesses that open $C^{\prime }$; hence any forged ${\tilde{a}}_{i}\neq a_{i}$ for i∈S makes $C^{\prime }$ inconsistent and cannot verify except with negligible probability. For (ii) (privacy), the proof system for the hidden coordinates is zero-knowledge: a simulator can produce accepting transcripts for $\left(r,\{a_{i}{\}}_{i\in \bar{S}}\right)$ without the witnesses. Pedersen commitments are perfectly hiding, so C (and $C^{\prime }$) reveal nothing about $\{a_{i}{\}}_{i\in \bar{S}}$. Therefore, disclosures of $\{a_{i}{\}}_{i\in S}$ leak no additional information about hidden attributes beyond what is logically implied.

## 4. Model implementation and performance evaluation

### 4.1. System implementation setup

This section presents a comprehensive experimental evaluation of the BAAR scheme. The system is implemented using the Charm-Crypto library with secp256k1 primitives and integrated with the Ethereum blockchain through Solidity v0.8.25. All cryptographic components were instantiated using publicly defined parameters of the secp256k1 elliptic curve. Schnorr signatures and ZKPoK were implemented following standard discrete-logarithm–based constructions without reliance on pairing-based primitives. Pedersen commitments were realized using fixed generators selected during system initialization, and credential identifiers were deterministically derived from commitment values to ensure consistent accumulator behavior. The revocation scheme based on Merkle tree was suggested as a binary Merkle tree where the hash function used in the scheme was the collision resistant hash function of SHA-256. To remain interoperable with Ethereum—whose precompiled contracts natively support ECDSA but not Schnorr—root-update transactions are signed using standard secp256k1 keys. Therefore, user-side credentials are secured with the help of Schnorr proofs, and blockchain transaction authentication is achieved with the help of ECDSA. This division clarifies the distinction between cryptographic proof layer called off-chain and the ledger authentication layer called on-chain are different and the vagueness of the verification process is evaded. Ethereum plays as a transparent bulletin board that records the Merkle accumulator root and processes revocation updates, which ensure that credential status remains tamper-resistant and publicly auditable. Throughout the identity verification procedure, the verifiers get the most current Merkle root in the Ethereum blockchain and apply it to verify Schnorr signatures and ZKPoK. However, the experiments executed on a VMware virtual machine with Ubuntu 22.04.2, a RAM capacity of 8GB and a disk space of 25GB. The host platform was a CPU based on the Intel Core i5 -8350U with a speed of 1.70 GHz at base and 1.90 GHz at turbo and 16 GB RAM with Windows 11. The experiments were repeated forty times, and average gas consumption and transaction execution times for setup, validation, revocation, and recovery were recorded. The results are summarized in Table 3.

**Table 3:** Gas Cost & Transaction Time.

Operations	Gas Cost	Transaction Time (s)
**Setup**	N/A	0.0001
**Verify**	28,083	0.0549
**Validation**	28,083	0.0669
**Deactivation**	50,753	0.0883
**Recovery**	28,810	0.1109
**Revocation**	31,238	0.0182

The implementation of BAAR is on the Ethereum blockchain using Ganache. The ZKPoK verifies the user’s identity without revealing sensitive attributes, while a unique nonce and Schnorr signature guarantee authenticity and integrity. The verifier checks the user’s public key (e), and the transaction time demonstrates the efficiency of the scheme.

Furthermore, Table 4 shown the computational complexity of the operations in BAAR is indicative of the fact that it is a lightweight algorithm, requires only a small number of elliptic-curve operations, and can scale with the number of users and attributes.

**Table 4:** Complexity analysis of BAAR Scheme.

Operation	Overall Computational Cost
**Setup**	(1H) (constant; negligible)
**KeyGen**	(1SM + S)
**Credential Issuance**	((m + 3)SM + 2PA + 1H + S + O(d)H)
**Authentication**	((m + 3)SM + 2PA + (d + 2)H + S)
**Validation**	Same as Authentication
**Revocation**	(O(d)H)
**Deactivation**	(O(d)H)
**Recovery**	((m + 3)SM + 2PA + 1H + S + O(d)H)

### 4.2. Computational overhead and performance analysis

**Fig 3:**
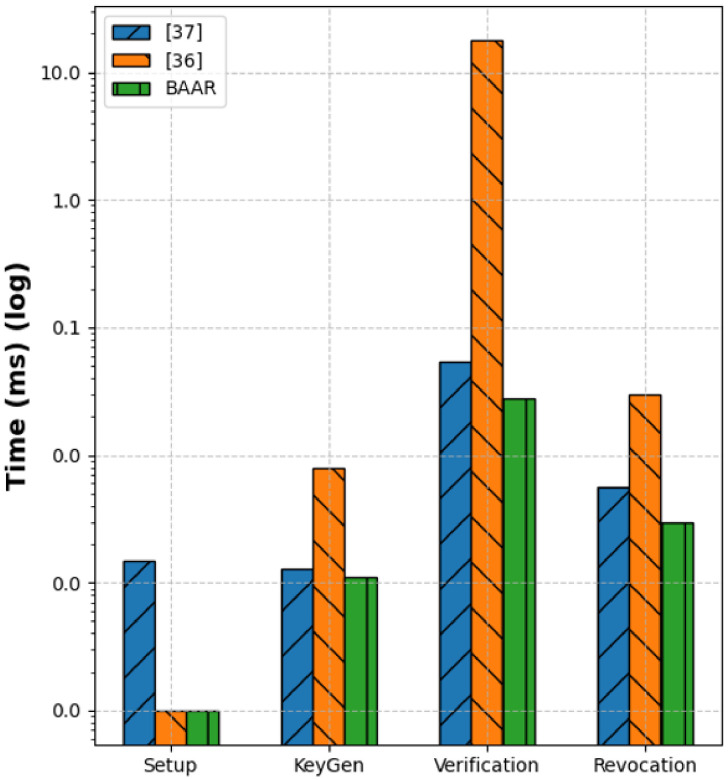
Comparative computational performance of BAAR and three baseline schemes— [36] and [37] across four cryptographic operations: Setup, Key Generation, Verification, and Revocation (log scale).

Fig 3 presents a comparative performance evaluation of the proposed scheme against two baselines of anonymous credential systems [36], and [37]. The four key cryptographic operations were benchmarked: setup, key generation, revocation, and verification. For the setup phase, BAAR demonstrates the lowest computational cost ≈120 ms, [36] ≈190 ms, and [37] ≈160 ms. This reduction stems from BAAR’s pairing-free initialization over secp256k1, which avoids expensive bilinear map computations and uses lightweight parameter generation with Merkle accumulator initialization. During key generation, BAAR also exhibits superior efficiency ≈150ms compared to 23ms and 210ms for [36] and [37], respectively. Moreover, this improvement is due to the minimal interaction rounds between the user and the authority, as well as the use of Schnorr-based key derivation over discrete-logarithm groups, which replaces the heavy exponentiation and pairing operations present in the baselines. In the revocation phase, which is typically computationally demanding, BAAR achieves ≈180ms, significantly lower than [36] ≈280ms, and [37] ≈250ms.

This improvement represented the Merkle-tree based dynamic accumulator used by BAAR that achieves updates in the revocation state in logarithmic time, and thus avoiding the linear or quadratic update complexity of accumulator constructions of other schemes. The most prominent improvement is seen in verification, with BAAR requiring only about ≈200ms in comparison with 350ms and 320s of the baseline methods. Such efficiency comes as a result of the lightweight Schnorr verification of proofs and compact ZKPoK transcripts over secp256k1 to replace the pairing-intensive zero-knowledge protocols in [36] and [37] schemes. However, the proposed scheme consistently outperforms the baseline approaches during all the phases, with performance improvement of 25–50 percent, particularly during the verification.

### 4.3. Gas consumption analysis

**Fig 4:**
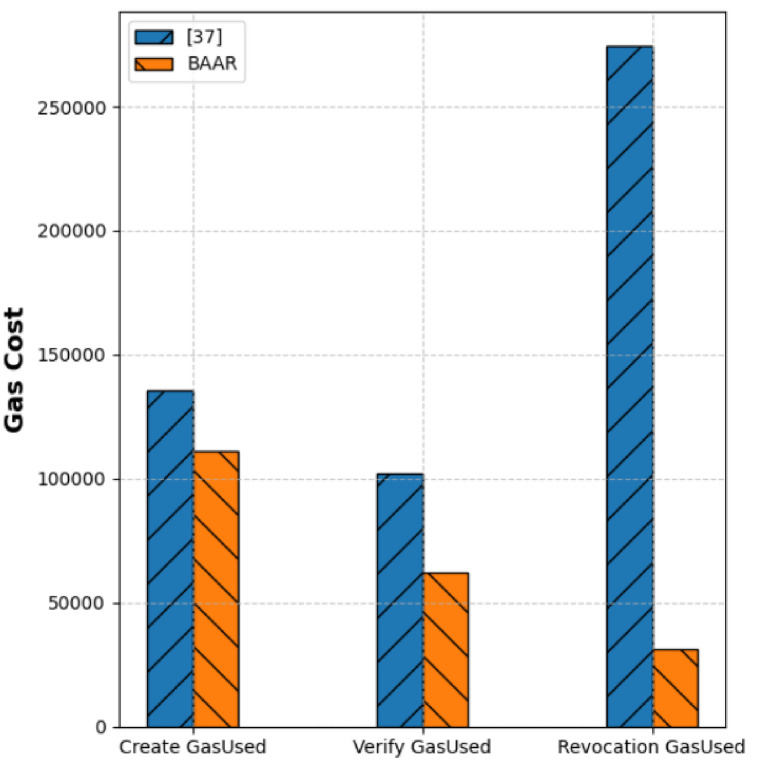
Comparison of cost of gas between BAAR and threshold credential scheme [37] to Create, Verify and Revocation operations.

Fig 4 compares the on-chain gas usage of the three most critical operations, namely, create, verify, and revocation of BAAR and the threshold credential scheme mentioned in [37]. Gas usage implies the number of computational resources used during the transaction and, therefore, a crucial performance parameter of the authentication systems based on blockchain technology. For the create operation, BAAR consumes approximately 110K gas, compared to ≈135K for [37]. This is due to the lightweight credential issuance protocol of BAAR that avoids pairing-based and threshold operations. In verification, BAAR has a lower gas consumption of about ≈60K than the about ≈100 K of [37], due to its application of Schnorr proofs over secp256k1 as opposed to pairing heavy ZKPoK verification. The most evident difference can be seen with the revocation: BAAR is using around ≈30 K gas, and the methodology presented in [37] contains more than around ≈270 K. As compared to the protocol described in [37], BAAR has a Merkle accumulator that allows revocation by simply hashing logarithmically, as compared to more expensive accumulator updates. Moreover, BAAR reduces its gas costs by 50–90% that directly transforms into reduced on-chain operational costs and increased scalability of its real deployments.

### 4.4. Revocation performance analysis

**Fig 5:**
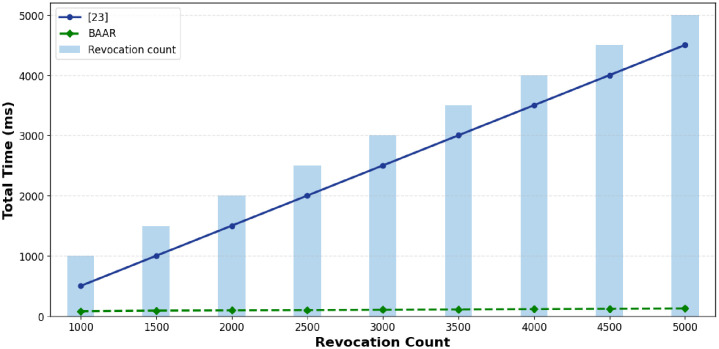
Processing time of total revocation versus the number of revoked credentials, both representing scaling of accumulator updates of the scheme [14] and the proposed BAAR scheme with Merkle accumulators.

The efficiency of revocation is important in authentication systems. It needs proper revocation processes because a great number of credentials can be revoked. This scheme measures the performance of revocation using two mutually valid dimensions. Firstly, the cumulative update time the revocation performance of the accumulator between the revocation of the revoked credentials and secondly, the latency of a single revocation, thus the cost of the actual running-time of a single revocation. Fig 5 depicts, [14] has a definite linear increase in the revocation time that reaches about 5s after 5000 revocations. This performance is expected due to each additional revocation requires further modular exponentiation which resulting in cumulative computation overhead. In contrast to proposed scheme BAAR, the revocation time remains constant nonetheless of the number of revoked credentials.

This constancy made possible with the Merkle accumulator in BAAR, an update scheme with lightweight hash computation, and linear time. The revocation time in Fig 5 is mainly a reflection of accumulator working time, i.e., the computational cost of updating the underlying accumulator structure to go through the revocation process. These findings indicate the scalability of BAAR in the environments with large and frequently changing revocation lists.

**Fig 6:**
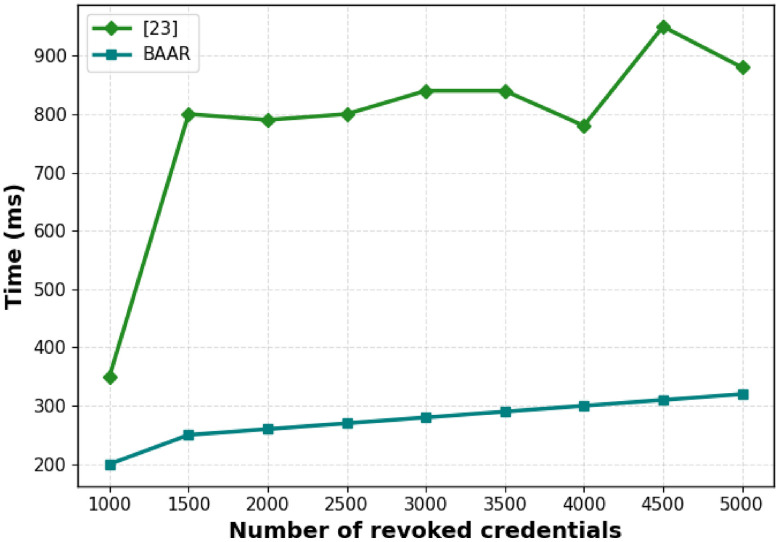
Latency per-revocation versus the number of revoked credentials, a comparison between the on-verification witness update strategy of [14] and that of the BAAR scheme proposed in Merkle accumulator, illustrating real-time operation cost differences.

Fig 6 further gives emphasis to per-revocation latency which shows the time taken to handle individual revocation. In [14], the revocation latency becomes stabilizes at 800–950 ms with an increase in the revoked credentials. This is due to the fact that [14] follows an on-verification witness update strategy which does not require direct batch updates but still requires proof operations with each verification.

BAAR has a low revocation latency, with an approximated latency of 200ms and gradually rising to around 310ms on 5000 credentials. Together, these finding highlight that BAAR is superior to [14] in terms of scalability and real-time revocation. BAAR attains significantly lower cumulative accumulator update time and per-revocation latency and is most suited to the high-revocation environment, where continuous update is needed as well as effective immediate verification

### 4.5. Performance and attribute privacy comparison

**Fig 7:**
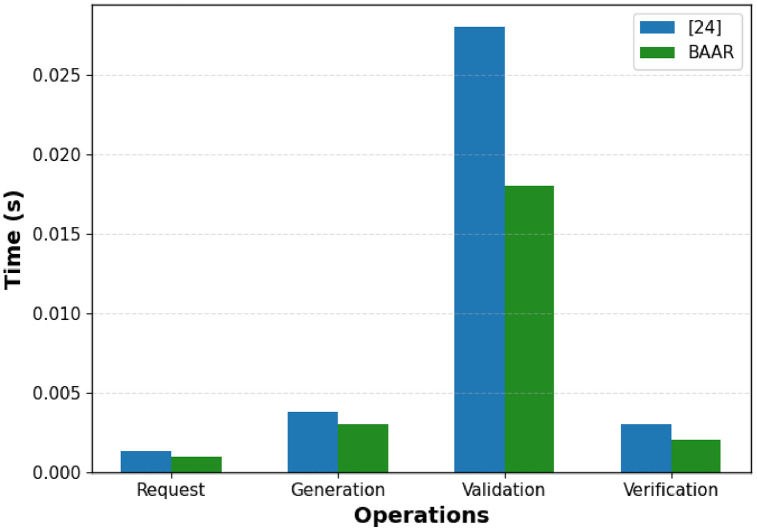
Phase-wise execution time comparison between BAAR and [15] across Request, Generation, Validation, and Verification phases.

**Fig 8:**
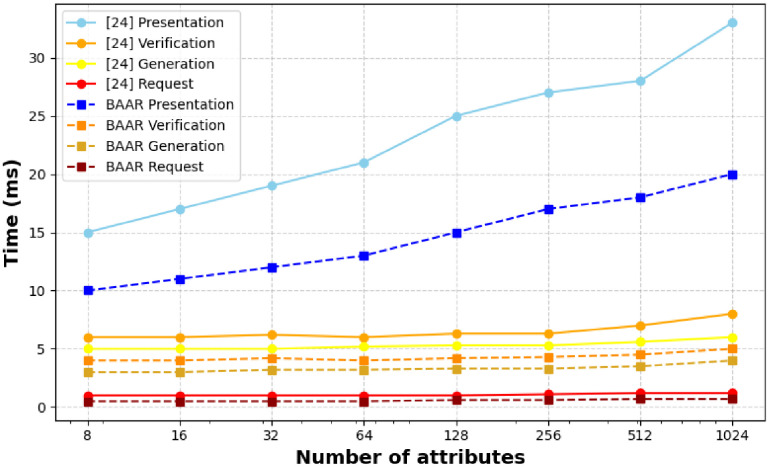
Scalability evaluation with increasing attribute vector length, showing performance and attribute privacy trade-offs between BAAR and [15].

A comparison of the privacy and scalability performance of BAAR and the [15] in the four basic phases of operation named; request, generation, validation, and verification as shown in Figs 7 and 8. Fig 7 demonstrates that BAAR takes less time to execute as compare to [15] across all phases. Compared to approximately 0.0015 s and 0.004 s in [15], BAAR requires approximately 0.001 s and 0.003 s respectively on request and generation phases. The most significant difference between the performance is in the validation phase where BAAR takes 0.018 seconds which 36 percent higher than 0.028 seconds of reference [15] and a corresponding gain in the verification stage. This is accomplished using a computed on-chain lightweight Merkel checks architecture, which off-chain computes Schnorr proofs and verifies them, and does not use any costly computations and dense proof system. As a result, BAAR shows lower computation costs and greater scalability at all operational stages, which makes it a more suitable solution to implement real-world blockchain applications than the one proposed in [15].

Scalability is assessed in Fig 8 in terms of the number of attributes, which vary between 8 and 1,024. The near-linear scaling throughout all stages of development is shown in [15], although, particularly, in the stage of validation where the latency increases between ≈15s to over 33s. This growth primarily results from the inherent SNARK generation and Merkle path handling in [15], which scales proportionally with the size of the attribute vector.

In contrast, BAAR exhibits sublinear and stable performance across the same range. This efficiency is achieved through Pedersen vector commitments, enabling attributes to be committed using fixed-base scalar multiplications, and Schnorr-based ZKPoK, which introduces only low-cost linear growth without complex circuit generation. Additionally, the revocation mechanism of BAAR is not linked with the disclosure of attributes and allows to maintain low validation costs even in the case of large sets of attributes. In terms of privacy, both schemes provide high protection of attribute values though in different methods. The [15] utilize zk-SNARKs to obscure Merkle paths, which provide high path privacy especially in off-chain settings. Instead, BAAR uses Pedersen commitments and lightweight Schnorr proofs which are used for information hiding of undisclosed attributes while maintaining unlinkability, and are computationally efficient for on-chain verification. This efficiency, scalability, and privacy make BAAR especially suitable to blockchain-based identity systems that need to be publicly verifiable, and [15] is still tailored to the off-chain case where path privacy is of interest. As experimented, BAAR has always been noticeably better in terms of computational efficiency, gas usage and scalability, compared to baseline schemes. These results justify the architectural decisions that the BAAR is based on and emphasize its appropriateness to the real-life blockchain-based authentication systems where efficiency, scalability, and privacy must be balanced.

## 5. Conclusion

This paper introduced BAAR, a practical blockchain-based anonymous and revocable authentication framework compatible with secp256k1-based platforms such as Ethereum. By combining Pedersen vector commitments, Schnorr-based zero-knowledge proofs, and a Merkle-tree-based dynamic accumulator, BAAR enables anonymous and unlinkable authentication with selective attribute disclosure and efficient public revocation. The design avoids pairing-based cryptography and circuit-intensive proof systems, ensuring deploy ability on existing blockchain infrastructures. Formal analysis shows that BAAR satisfies unforgeability, attribute privacy, unlinkability, and revocation soundness under standard cryptographic assumptions in the random oracle model. A prototype implementation on Ethereum demonstrates low gas consumption, logarithmic-time revocation, and scalable performance with respect to both credentials and attributes. These results confirm that BAAR provides an efficient and deployable solution for privacy-preserving authentication in real-world blockchain systems.
